# A Cross-Continental Study on Children's Drawings of Football Players: Implications for Understanding Key Issues and Controversies in Human Figure Drawings

**DOI:** 10.5964/ejop.v13i3.1237

**Published:** 2017-08-31

**Authors:** Bahman Baluch, Linda J. Duffy, Rokhsareh Badami, Elisangela C. Ap Pereira

**Affiliations:** a School of Science and Technology, Middlesex University, London, United Kingdom; b Department of Physical Education and Sport Science, Isfahan (Khorasgan) Branch, Islamic Azad University, Isfahan, Iran; c Barao de Maua University, Ribeirao Preto, Brazil; Webster University Geneva, Geneva, Switzerland; The Maria Grzegorzewska University, Warsaw, Poland

**Keywords:** children's drawings, football, cross-continental, human figures

## Abstract

Professionals examine various aspects of girls’ and boys’ drawings as a way of understanding their intelligence, personality and emotional state. However, the extent to which such measures could be universally generalised or attributed to a specific cultural norm is still a debatable issue. In the present study five key features of children’s drawings namely: the size (height) of the drawings, profile or full face, figure in action or static, shaded or non-shaded and the nature of additional details were examined from a cross-cultural perspective, and by providing a topic (football) for which children’s drawing of a human figure could provide opportunities for the latter indices to manifest and flourish. Children from three countries; England, Iran and Brazil, representing three continents took part in this study. The participants were asked to draw a football player from their own country and from the other participating countries. The results showed that Brazilian children differ from Iranian and English children by drawing significantly smaller figures and putting more football action in the drawings. Shading of the figure drawn was more prevalent amongst English children. Such findings have implications for the interpretation of key aspects of children's drawings in educational, clinical and therapeutic settings and from a universal vs. culturally-specific viewpoint.

Children’s drawings can be understood as “a mirror to their minds” and providing access to the child's representational work (Cherney, Seiwert, Dickey, & Flichtbeil, 2006). Considerable systematic research dating back to the beginning of the 20th century (e.g. Rouma, 1913; Schuyten, 1904) has been aimed at evaluating and interpreting children's attempts in producing some form of "drawings", ranging from scribbling on a page to a more conventional form (see Cox, 1992; Golomb, 1992 for reviews). In particular, interest concerns children’s depictions of human figures and especially drawings of themselves and others. It has been argued that this is due to the extent of importance of human beings to the child from a very young age and the most frequently drawn topic by children, thus a better index for understanding the child's cognitive (intellectual) and emotional development (Cox & Hodsoll, 2000). One significant and consistently observed aspect of children's drawings is that with increasing age there is an increase in the addition of realistic portions of the human figure. This led to the popularly used Draw-a-Man test originated in 1926 by Goodenough, later modified by Harris (1963) and Koppitz (1968) and the stage theories of developmental aspects of children's drawings (see Golomb, 2002). The task is to simply ask a child to draw a man (later modifications to draw a man and a woman) and to examine an accumulative count of realistic body parts from a prescribed list (e.g. head, eyes, arms). The list varied from 51 items in Goodenough's (1926) original list to Harris’s (1963) 71 items and a more compact 30 item list by Koppitz (1968). As the score obtained was shown to correlate significantly with standard IQ tests (Koppitz, 1968) it gave rise to using the Draw a Man test as a valid and un-intrusive measure of a child's intellectual ability (Hartley, Somerville, Jensen, & Eliefja, 1982). Furthermore, it was argued that the Draw a Man test could be as close as we have come to an ideal, culture-free test of intelligence (Di Leo, 1970). For more recent reviews see Imuta, Scarf, Pharo, and Hayne (2013).

A further development into the assessment of children's drawings was made when it was noted that inferences could be made by the nature of the drawings as well as the method of counting items drawn (Koppitz, 1968). For example, an exaggerated size of the head or body, shading, inclusion of extra items such as clouds, rain, snow, flying birds or by cutting portions of the arm. Koppitz (1968, 1984) argued that such depiction by children reflects their feelings, fears, dislikes and anxieties. This gave rise to the development of a 30 item emotional indicators by Koppitz (1968) as an attempt to assess a child's emotional adjustment and disturbance.

Several reported studies have considered Koppitz’s (1968) emotional indicators as a tool to assess their clinical and non-clinical populations (e.g. Lee & Hobson, 2006; Nyman, Baluch, & Duffy, 2011; Nyman, Baluch, Duffy, & Shinebourne, 2011, see also Skybo, Ryan-Wenger, & Su, 2007 for a review). In a similar fashion children’s human figure drawings have been of interest to therapists not only for assessment and diagnosis but also as a focus of the therapy process to interpret and assess for signs of children’s feelings about people and events in their lives (Deaver, 2009, Pala, Nuvvula, & Kamatham, 2016).

## Factors to be Taken Into Account When Assessing Children’s Human Figure Drawings

In spite of the great appeal for evaluating children’s human figure drawings, it has now been noted that a number of factors, both in educational and clinical practice, have to be taken into account before a correct assessment is made. In educational terms a strictly Piagetian description of the stages of development of children's drawings (Piaget & Inhelder, 1956) has been challenged by considering alternative accounts, namely task demands and cue‐dependency (Burkitt, 2017). In particular, the impact that a communicative context may have on children’s expression of mood in drawings of inanimate and animated topics. Similarly, there has now been realisation that factors such as the significance that the drawing has for the child could have their own independent effect on how and why children draw a human figure. For example, studies have shown that when children were asked to draw themselves and family members there is a significant change in how children may portray a person, such as putting more action and movement in the drawing rather than portraying a static person (Burns & Kaufman, 1970). Similarly research by Cherney et al. (2006) has reported that when children were asked to draw their family and their school there was a significant gender and age difference in the number of details depicted in the family drawings. However, one noticeable factor that could play a significant role in children’s human figure drawings is the cultural factor. It is now clear that in spite of the early excitement that interpretation of children's drawing could be a culture-free test of abilities and emotions, there has nevertheless been acknowledgment that cultural difference could influence children's drawings (e.g. Kellogg, 1970; Kellogg, 1979; Wilson & Wilson, 1984). This is because it has been argued that the drawing of a human figure may not mean the same thing to children from different ethnic and cultural groups (Court, 1989, 1994; Hui & Triandis, 1985). In particular, with respect to aspects of Koppitz (1968, 1984) emotional indicators such as the size of the drawing, shading of the drawing, drawing a profile rather than a full face and including the amount of details, there are concerns about the extent to which such drawings could be affected by cultural factors and therefore could be the subject of misinterpretation (Cox, 1992). For instance, most research on Western children reports that they draw a full face when asked to draw a human figure rather than a profile (see Dziurawiec & Deregowski, 1992; Pinto & Bombi, 1996). Most Western children prefer to draw a human figure looking static rather than engaged in an action (Cox, 1993). Moreover, it may depend on what is required from the child to draw (e.g. a person doing something) or age and the gender of the child (Cox & Lambon Ralph, 1996; Lark-Horovitz, Lewis, & Luca, 1973). Cross-cultural studies have shown that indeed there are differences in how children from different cultural groups decide to draw a profile or full face. For example, Cox, Koyasu, Hiranuma, and Perara (2001) reported significant differences in how children from the UK and Japan draw human figures. Similarly, another aspect of children's drawings, namely the size of the drawing, has been the subject of investigation within and across different cultural groups. Richter (2001) reported significantly smaller figure sizes of preschool-aged Madagascan Mahafaly children when compared to German children.

One other interesting aspect of children's human figure drawings which is also included in Koppitz (1968) emotional indicators is whether children shade their drawings e.g. depicting a person from a non-white race. This is because children most frequently draw a white person even if they are from a non-white ethnic group. For example, Dennis (1966) found that children from Cambodia, Greece, Iran, Israel, Japan, Lebanon, Mexico, Sweden, Taiwan, Turkey, UK, USA and Germany drew human figures that appeared as a white man in Western dress. Similarly, Papadakis-Michaelides (1989) found that among the 3200 figures drawn by 1600 children from five cultural backgrounds in the UK (English, Hindu, Muslim, Sikh and West Indian) aged 3 years 6 months to 11 years 6 months only one figure was dark skinned. Also girls in the latter study generally drew taller human figures than did the boys. This discrepancy could be largely due to the extent to which the drawing plays a significant role in the child's life. Children from the Dennis (1966) or Papadakis-Michaelides (1989) studies may be thinking of depicting a white person because they may think this is what is required of them. This could also be an example of how communication context may affect children’s drawings (Burkitt, 2017). The children would have drawn a person with a dark skin if they were asked to draw a specific person of interest that may not be Caucasian. Furthermore, the size of drawings may be more related to the level of interest and significance that a child may attach to the specific drawn person rather than simply a gender factor. Fox and Thomas (1990) found that most children draw their parents taller than ordinary people. In a similar fashion the level of interest and significance that a child may attach to any topic may vary in different cultures. Cox (1992, 1993) has argued that it is a challenge to find a topic that is of the same level of interest across different cultures and for this reason children from various cultural and ethnic backgrounds may draw a human figure in very different ways for different underlying reasons.

## Aims of the Study

In view of such discrepancy it was considered to identify a topic that would be of particular interest to children, particularly boys, worldwide that included plenty of known personalities of various ethnic backgrounds. The reason that the topic was selected to be of particular interest to boys was that it has often been claimed that girls show more interest in arts and boys in science (Murphy, 1997). Thus girls are said to be more interested in drawings than boys, use more colours than boys and “boys just aren’t interested” Murphy (1997, p. 122). The topic of drawing a football player should provide an ideal opportunity for boys to manifest their abilities, creativity, hidden talents and skills in human figure drawings. It is in this context that some of the classic findings and interpretations of children’s drawings such as gender differences, size of drawings, shading or not shading, drawing in profile or additions of context could be given a different interpretation and may be looked at in terms of what is normal and what is deviation from the norm; providing perhaps valuable clues when evaluating children’s human drawings by clinicians, educationalists and therapists (Deaver, 2009).

However, as the topic selected for the present research is human figure drawings of a football player it would be necessary to address briefly the development of sport participation, particularly for the children of the age group targeted in the present study. It has been argued that children are both qualitatively and quantitatively different from adults in maturation and their experience and sport participation which may result in various developmental outcomes (Wiese-Bjomstal, LaVoi, & Omli, 2009). Taking into account Piagetian description of the stages of development it is clear that the manner that very young children (below the age of 7) may regard sport participation and behaviour is very different from older children. For example, very young children may not see the same football scene being played from various angles and perspectives. With increasing maturity and, in particular, between the ages of 7-11, a stage known as the “Concrete Operational Period” they have developed a greater intellectual ability to represent transformations in terms of their sporting interest. Thus by targeting a population of children at this critical stage of their maturational development one could further examine how their drawings of human figures in the format of a football player may represent transformations in terms of their sporting interest.

In view of the above arguments, the present study aimed to examine whether there are cultural differences on the following key factors in children's human figure drawings, namely size of the drawings, shading or not shading, amount of detail and whether or not they are static or showing movement. We also examined gender differences when boys and girls (aged between 9 and 10) from three successful footballing nations were asked to draw a football player from their own country and from two other countries across different continents. Football is a topic that is of interest worldwide to all age groups, thus it provides a great opportunity to examine the extent to which the above features are universal or culture-specific. The three countries selected were Iran, Brazil and England. The latter two countries are universally known for their development of the game and the level of enthusiasm that they have shown in the game. Football is also one of the most popular sports in Iran where the National team has been ranked as the top Asian nation for the past decade.

## Key Questions

Will there be significant differences in the size of drawings of football players, the use of profile in drawings and the action put into the drawings between the children of the three participating countries and between boys and girls? Furthermore, the present study will examine if there will be significant difference between the children from the three participating countries and between boys and girls on the number of figures drawn shaded or not shaded? Other factors investigated are whether the children from the three participating countries differ significantly in how much detail (e.g. adding a name, other players, or spectators) they add to their drawings and between boys and girls?

## Method

### Participants

Overall 196 children from three countries representing three continents, namely South America, Asia and Europe, took part in this study. Brazil (*N* = 71, 30 girls *M* = 9.44, *SD* = 0.68, and 41 boys *M* = 9.73, *SD* = 0.83). Iran (*N* = 81, 24 girls *M* = 10.54, *SD* = 1.14 and 57 boys, *M* = 10.54, *SD* = 1.31) and England (*N* = 44, 17 girls *M* = 10.94, *SD* = 0.24 and 27 boys, *M* = 10.85, *SD* = 0.36). All children were selected from the populations’ middle class with no known emotional or psychological disorders.

### Materials and Procedure

Permission and ethical approval for the study was sought from Universities where the authors are employed and from the schools that agreed to permit access to the pupils. Data was collected by three contributors to this study, natives from the following countries Brazil, Iran and England and data collection was carried out in their respective countries.

The procedure for data collection was identical for all three locations. Each child who took part in this study was given parental consent and agreement that the drawings may be used anonymously in a subsequent publication(s). The study was also conducted during convenient times for the schools and as part of their routine art classes. Art education is a focal aspect of the curriculum in Brazil, Iran and England. For example, in Brazil since 1971, art education is a compulsory subject at schools (Barbosa, 2015). Similarly, in Iran there is a great emphasis on art education, particularly drawings, as part of the school curriculum (Rooholamini, 2001). See also Department for Education (2013) publication for detailed accounts on art education in English schools.

Each participant was approached by the researcher and was given an A4 size paper and a set of coloured and black pencils and asked to draw a football player. The choice of country to draw the player was selected randomly on each occasion e.g. a Brazilian child was asked to draw a football player from England followed by a player from Brazil and finally from Iran. The second participant was asked to draw first a Brazilian player followed by an Iranian player and finally a player from England and so on. This was to avoid any order effects in their drawing strategy (see van Sommers, 1989). The children were then thanked for their participation in the study. The procedure for scoring each drawing was conducted by the main researcher in each of the three countries. Guidelines were provided for each researcher regarding how to score the key aspects of the drawing namely size, profile, and the amount of details, shading and action. Furthermore, the key researchers in each country exchanged examples of drawings and their scoring to ensure reliability of scoring and to avoid any bias.

## Results

Data was analysed separately as per country and gender of the child with respect to the following key aspects of the drawings: namely size (height) of human figures in centimetres, whether or not the drawn figure was full face or a profile, whether or not the drawn figure was static or showing action, whether there were additions other than just a human figure and finally whether or not the figure drawn was shaded (i.e. depicting a non-white person).

### Descriptive Statistics

As can be seen in Table 1, Brazilian children have drawn the smallest size of football players followed by the Iranian children, the largest football players were drawn by children from England. Also, Brazilian children seem to use more profile and action in their drawings of football players followed by children from England and the Iranians seem to use the least profile in their drawings. Furthermore, Brazilian children as a whole have more additions in their drawings than children from England and Iran. Children from England had used shading more extensively than Brazilian and Iranian children who hardly used any shading in their drawings. Overall, males had used more shading than females.

**Table 1:** Mean and Standard Deviations as per Condition in the Present Study

Participants/Gender	Male Player drawn						Female Player drawn					
	Brazil		Iran		England		Brazil		Iran		England	
	*M*	*SD*	*M*	*SD*	*M*	*SD*	*M*	*SD*	*M*	*SD*	*M*	*SD*
Brazil (size)	8.79	4.30	7.76	3.50	7.80	3.80	9.43	4.20	8.55	3.90	9.04	4.62
Iran (size)	10.53	5.51	11.13	6.37	10.75	6.20	11.26	4.60	11.09	4.60	9.89	4.40
England (size)	13.25	5.34	13.85	6.29	13.81	6.56	12.25	7.06	11.68	5.55	12.50	5.57
Brazil (profile)	0.21	0.41	0.09	0.33	0.04	0.21	0.13	0.34	0.03	0.18	0.06	0.25
Iran (profile)	0.00	0.00	0.00	0.00	0.01	0.13	0.00	0.00	0.04	0.20	0.04	0.20
England (profile)	0.30	0.19	0.03	0.19	0.03	0.19	0.00	0.00	0.00	0.00	0.12	0.34
Brazil (action)	0.48	0.50	0.39	0.49	0.39	0.49	0.26	0.44	0.43	0.50	0.36	0.49
Iran (action)	0.18	0.39	0.22	0.41	0.14	0.16	0.12	0.33	0.25	0.44	0.37	0.50
England (action)	0.44	0.50	0.03	0.19	0.40	0.50	0.25	0.44	0.06	0.25	0.37	0.50
Brazil (additions)	0.58	0.50	0.53	0.50	0.48	0.50	0.48	0.50	0.48	0.50	0.44	0.50
Iran (additions)	0.12	0.33	0.14	0.35	0.09	0.29	0.12	0.33	0.25	0.44	0.20	0.41
England (additions)	0.03	0.19	0.07	0.25	0.11	0.32	0.18	0.43	0.25	0.44	0.25	0.44
Brazil (shading)	0.48	0.21	0.04	0.21	0.00	0.00	0.03	0.18	0.00	0.00	0.00	0.00
Iran (shading)	0.00	0.00	0.00	0.00	0.00	0.00	0.00	0.00	0.00	0.00	0.00	0.00
England (shading)	0.59	0.50	0.51	0.50	0.55	0.50	0.18	0.40	0.25	0.44	0.18	0.40

*Note.* Mean size of each drawing in centimetres, mean number of drawings that were drawn as a profile, mean number of drawings that showed action, mean number of drawings that had additions included, and the mean number of drawings that used shading together with their corresponding standard deviations (in brackets) as per country of the participant and gender.

### Size (Height)

A 3 x 2 x 3, Country (Brazil, Iran, England) x Gender x Player (Brazil, Iran, England) mixed factorial ANOVA was conducted on size of drawings in centimetres. There was a significant main effect for country with *F*(2, 185) = 10.54, *p* < 0.001, *MSE* = 70.11. There was no significant main effect for gender *F*(1, 185) = .087, *p* = 0.77, *MSE* = 70.11 and no significant interaction effect with *F*(2, 185) = 0.77, *p* = 0.46, *MSE* = 70.11. Post-hoc comparisons of the means for country using Tukey's LSD showed a significant difference for size of drawings between children from Brazil and Iran with *M* = 2.29, *SE* = 0.79, *p* = 0.004, children from Brazil and England with *M* = 4.58, *SE* = 0.93, *p* < 0.001, and children from Iran and England with *M* = 2.29, *SE* = 0.92, *p* = 0.014.

### Profile

A 3 x 2 x 3, Country (Brazil, Iran, England) x Gender x Player (Brazil, Iran, England) mixed factorial ANOVA was conducted on the number of times that a profile was drawn by children. There was a significant main effect for country with *F*(2, 186) = 5.39, *p* = 0.005, *MSE* = 0.068. There was no significant main effect for gender *F*(1, 186) = .065, *p* = 0.80, *MSE* = 0.068 and no significant interaction effect with *F*(2, 186) = 0.85, *p* = 0.43, *MSE* = 0.068. Post-hoc comparisons of the means for country using Tukey's LSD showed a significant difference using profiles between children from Brazil and Iran with *M* = 0.09, *SE* = 0.02, *p* < 0.001 and children from Brazil and England with *M* = 0.06, *SE* = 0.02, *p* = 0.028, the difference between children from Iran and England was not significant *p* = 0.39. Indicating that Brazilian children draw more football players with their profile compared to children from England and Iran, with Iranians drawing the least profile.

### Action

A 3 x 2 x 3, Country (Brazil, Iran, England) x Gender x Player (Brazil, Iran, England) mixed factorial ANOVA was conducted on whether or not children had drawn the football player in action. There was a significant main effect for country with *F*(2, 186) = 6.2, *p* = 0.002, *MSE* = 0.35. There was no significant main effect for gender F(1, 186) = 0.76, *p* = 0.38, or interaction effect with *F*(2, 186) = 0.17, *p* = 0.84. Post-hoc comparisons of the means for country using Tukey's LSD showed a significant difference in shading between children from Brazil and Iran with *M* = 0.21, *SE* = 0.05, *p* < 0.001 and a near significant difference between children from England and Brazil with *M* = 0.12, *SE* = 0.06, *p* = 0.06, the difference between children from Iran and England was not significant *p* = 0.17.

### Additions

A 3 x 2 x 3, Country (Brazil, Iran, England) x Gender x Player (Brazil, Iran, England) mixed factorial ANOVA was conducted on whether or not additions other than a football player were included in the drawings. There was a significant main effect for country with *F*(2, 185) = 17.82, *p* < 0.001, *MSE* = 0.40. There was no significant main effect for gender *F*(1, 185) = 1.06, *p* = 0.30, *MSE* = 0.40 and no significant interaction effect with *F*(2, 185) = 1.05, *p* = 0.35, *MSE* = 0.40. Post-hoc comparisons of the means for country using Tukey's LSD showed a significant difference in adding context using profiles between children from Brazil and Iran with *M* = 0.35, *SE* = 0.06, *p* < 0.001 and children from Brazil and England with *M* = 0.36, *SE* = 0.07, *p* < 0.001, the difference between children from Iran and England was not significant *p* = 0.84 indicating that Brazilian children included more context compared to Iranian and English children.

### Shading

A 3 x 2 x 3, Country (Brazil, Iran, England) x Gender x Player (Brazil, Iran, England) mixed factorial ANOVA was conducted on whether or not children had shaded the drawings of the football players. There was a significant main effect for country with *F*(2, 186) = 55.68, *p* < 0.001, *MSE* = 0.11. There was a significant main effect for gender *F*(1, 186) = 16.26, *p* < 0.001, *MSE* = 0.11 and a significant interaction effect with *F*(2, 186) = 11.45, *p* < 0.0001, *MSE* = 0.11. Post-hoc comparisons of the means for country using Tukey's LSD showed a significant difference in shading between children from England and Iran with *M* = 0.42, *SE* = 0.03, *p* < 0.001 and children from Brazil and England with *M* = 0.40, *SE* = 0.03, *p* < 0.0001, the difference between children from Iran and Brazil was not significant *p* = 0.46. Analysis of simple effects comparing drawings by male and female children from England showed a significant effect with *F*(1,41) = 7.73, *p* = 0.008 indicating significantly more shading by male children from England than female children.

## Discussion

Key features in children's human figure drawings were examined that have been subject of extensive research and interpretation, namely size, profile, action, context and shading. Of particular interest was whether there are gender and cultural differences on these key features when the figure to be drawn is a football player. Football is a topic that is of interest worldwide to all age groups and may be argued to provide a basis for more convergence of thoughts when drawing a human figure.

As a whole the country of the participants was found to be the most significant variable in all comparisons, with Brazilian children drawing the smallest of the players, adopting significantly more profiles in their drawings, and adding more additions to the context upon which the player was drawn and, most interestingly, using more action for the player drawn. Children from England, however, drew more players that were seen to be shaded (implying non-white) than other participants. Gender differences showed significance only when analysed for shading, where boys from England drew more shaded figures than girls from England. Each aspect of the findings are now discussed in light of the relevant literature.

### Size

The absolute figure size of a person drawn is one of the most investigated features of human figure drawings with various interpretations. Koppitz (1968) in developing her criteria of emotional indicators in children's drawings paid particular attention to size as an indicator of emotions towards self and others, e.g. lower self-esteem, feelings of intimidation associated with smaller drawings of self. Gellert (1968) found that taller drawings by children are for people who seem to be more important or of interest to the child. In support of the latter notion, Fox and Thomas (1990) found that most children draw their parents taller than ordinary people. Research has also shown that other factors could also have an influence, for example, the size of a figure is reduced when additional figures or objects are drawn on the same page (Lange-Küttner, 2009; Thomas & Silk, 1990) and when a figure gets a negative connotation (Burkitt, Barrett, & Davis, 2005, 2009). However, these assumptions are based predominantly on the results of experimental studies and with participants mainly living in Western cultural environments. Few studies are reported on the size of drawings and cultural differences. The early research by Koppitz and de Moreau's (1968) study showed that Mexican children draw significantly smaller figures than children from the USA. However, the authors were not conclusive as to whether such differences were culturally based or due to social class, dietary or other forms of neurological deficits. Meili-Dworetzki's (1981) study found that Turkish children from Istanbul draw human figures a fourth smaller than do Swiss children of the same age. Here again the author attributed the differences to the socio-cultural context expressed in children's drawings. Payne (1996) reported that Caribbean, unlike children from the USA, drew their mothers taller than their fathers. Richter (2001) reported significantly smaller figure sizes of preschool-aged Madagascan Mahafaly children as compared to German children, and likewise, Rübeling et al. (2011) found 4-6 year-old Cameroonian children drawing themselves up to 55% smaller than German children from middle-class families. Recent studies on young children (up to age 6) compared children's drawings of themselves on the size and facial expression between Western urban (German and Swedish) and non-Western urban (Turkish, Costa Rican and Estonian) and non-Western rural (Cameroon and India). The results showed that children from Western and non-Western urban educated contexts drew themselves rather tall, with many facial features, and preferred smiling facial expressions, while children from rural traditional contexts depicted themselves significantly smaller, with less facial details, and neutral facial expressions (Gernhardt, Rübeling, & Keller, 2013, 2014). However, these studies are not very conclusive as to how children from different cultures would have drawn others in terms of size or additions to the drawings. In the present study the topic to be drawn was of interest to all participants, particularly boys, and the children tested were from three different continents all from the average sectors of the society and there were no indications of intimidation or dietary concerns.

**Figure 1:**
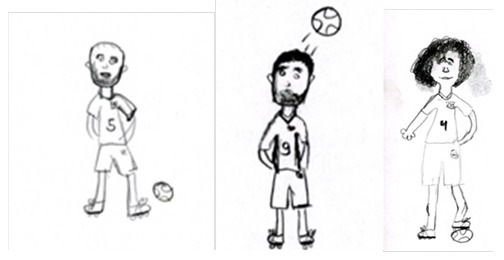
A Brazilian child's drawing of players from Iran, England and Brazil in their actual size.

The findings that Brazilian children draw significantly smaller figures of football players than children from England and Iran are of particular importance as it signifies culture as a factor to consider when evaluating size of human figure drawings insofar as drawings of others are concerned (see Figure 1). Brazilian children and the nation as a whole admire the game of football and their players, thus there is no reason that lack of interest or emotions attached are factors to draw smaller figures. Furthermore, the children in the study were all of the same socio-economic status as other participants in the study, thus the latter factors could be ruled out as a reason for their smaller drawings.

### Profile

**Figure 2:**
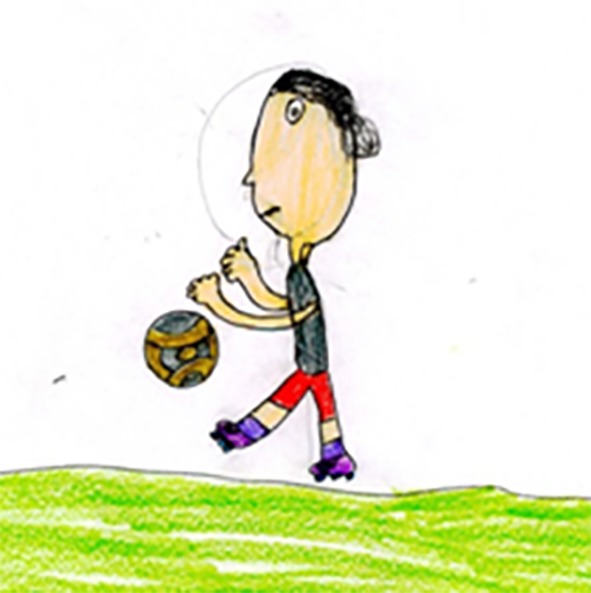
A Brazilian boy's drawing of a player from Iran depicting both profile and action.

A number of authors have noted that most children’s human figures are drawn canonically i.e. facing the viewer rather than in profile (Dziurawiec & Deregowski, 1992). According to Cox and Lambon Ralph (1996), Cox and Hodsoll (2000) with the increase in age between 5-10 years there is also an increase (nearly double) in the number of children who draw the profile of a human figure rather than full face. In particular this increase is greater if the children are asked to put action (e.g. a person running) into their drawings. The increase in age and shift in children's drawing of human figures is in line with Piaget and Inhelder's (1956) belief that very young children (below the age of 5 years) lack projective spatial concepts, thus will not represent the different views of objects, but with increasing age there is a shift from an internal model or idea of how a person is drawn to an externally presented model. The latter hypothesis was tested when children between 5 and 9 years of age were asked to look at a human figure (male model figure) in different positions, including running, and asked to draw a picture of this man (Cox & Lambon Ralph, 1996). The findings showed that only at the age of 6-7 years are most children able to adapt their human figure drawings successfully to take account of the model. However, there are reasons to believe that cultural differences may be a factor to consider, i.e. a late development in the ability to draw a human profile according to Western research may be challenged by finding that children in a different culture develop this ability much earlier. Golomb (2002), for example, reported higher incidence of profile forms in young Maori children’s drawings in New Zealand as compared with a relatively late occurrence in the drawings of the same age children from Europe and North America. In the present study when 9-10 year old children from three continents where asked to draw football players the frequency of profile drawing amongst Brazilian children was significantly more than the children from England or Iran. Arguably this suggests that factors other than the age and maturation may account for the ability to draw a human profile. One possible explanation for this could be the finding that Brazilian children also drew more players in action than static. As mentioned previously, when children were asked to draw a human figure in action the frequency of drawing a profile was significantly increased (Cox, 1993; Dziurawiec & Deregowski, 1992). Brazilian children seem to both draw football players that show more action and depict a profile rather than full face (see Figure 2).

### Action

According to Lark-Horovitz, Lewis, and Luca (1973), Cox (1993), Cox and Lambon Ralph (1996) boys in Western countries show interest in portraying movement and figures in action and girls pay more attention to static scenes with much detail and decoration. Furthermore, there is a developmental shift from static drawings to drawing figures in action with boys altering their rigid schemata of the human figure at an earlier age than girls. Two factors, however, should also be taken into account here; first when children are asked to draw in a context of someone doing something or to draw themselves and their family e.g. Kinetic Family Drawings (Burns & Kaufman, 1970) there is a significant shift in children portraying more valid and dynamic material than static pictures, moreover evidence of cultural differences emerge (Cox & Hodsoll, 2000). In a recent study Gernhardt, Rübeling, and Keller (2013) examined the family drawings of preschool-aged children from Western middle-class families from Osnabruck, Germany; from rural Cameroon and children from urban middle-class families from Ankara, Turkey. The family drawings varied with cultural context and the respective orientation toward autonomy and relatedness, specifically in regard to the number and position of family members, the depicted absolute and relative size of family members, the details of facial features, and the emotional expression. In the present study we simply asked the participants to draw a football player with no further instructions relating to movement. Of significant interest was that Brazilian children had significantly more drawings portraying a player in action than Iranian children or English children. Furthermore, there were no significant gender differences or gender by country interaction. This finding challenges the notion that there are differences in action or static drawing between boys and girls of the same age, even if the topic to be drawn is a football player. What is, however, interesting is the reason for Brazilian children portraying more action. As reported earlier, Brazilian children draw more human profile than Iranian children and English children, thus this contributed to depicting more action. Another reason might be that, perhaps in view of their great interest in the game of football, Brazilian children see the game more in action than static, a factor that may play a crucial role in why the nation has more football talent than most other countries.

### Additions

**Figure 3:**
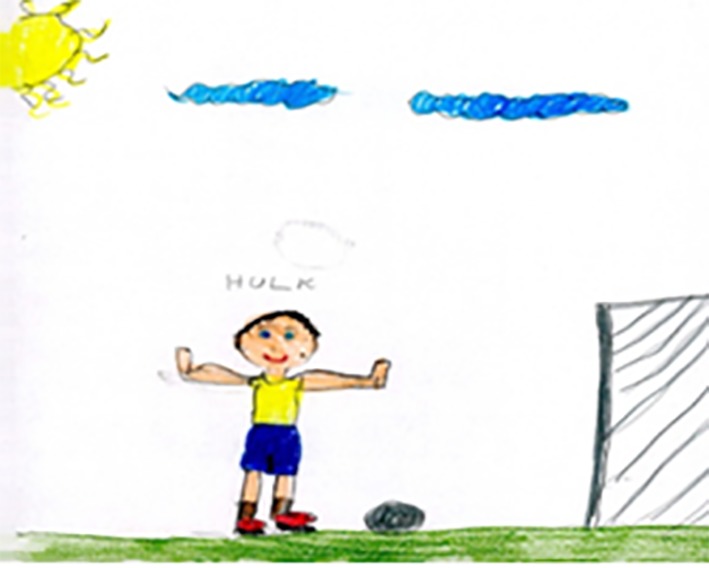
A Brazilian girl's drawings of a player from Brazil.

In most cases when children are asked to draw a human figure they do not include additions or background to the drawing, although according to Cox (1993) some elements are reported to be added after the drawing was completed. However, as mentioned earlier, when children are asked to complete a drawing that is not specific to just one person (such as the Family Kinetic drawings) one may see significant differences especially with regards to gender in terms of the number of additions to the background (Burns & Kaufman, 1970). Gender differences in what children may include in their drawings are also noticed from earlier studies. For example, Papadakis-Michaelides (1989) asked 240 children, aged between 4 years 6 months and 10 years 6 months, to draw a man and a woman doing something. The results showed that male participants are more likely to draw the person engaging in sport and females more likely to draw the person doing housework. In relation to cultural differences there has been no specific research to examine what children from different ethnic groups may add to their drawings if they are only asked to draw a person. In the present study, in spite of asking all children to draw a football player, there was a significant difference in how much additional context was included in the drawings. In particular, it was the Brazilian children who made significantly more additions, such as the presence of a goal post or the sun and clouds in the background (see Figure 3). Most interesting was that there were no gender differences in the amount of additions in all comparisons, although a few of the female participants tended to draw a female football player and boys predominantly drew a male player. One interpretation may be that Brazilian children see more in the game of football than children from Iran and England, as well as putting more action into their drawings.

### Shading

**Figure 4:**
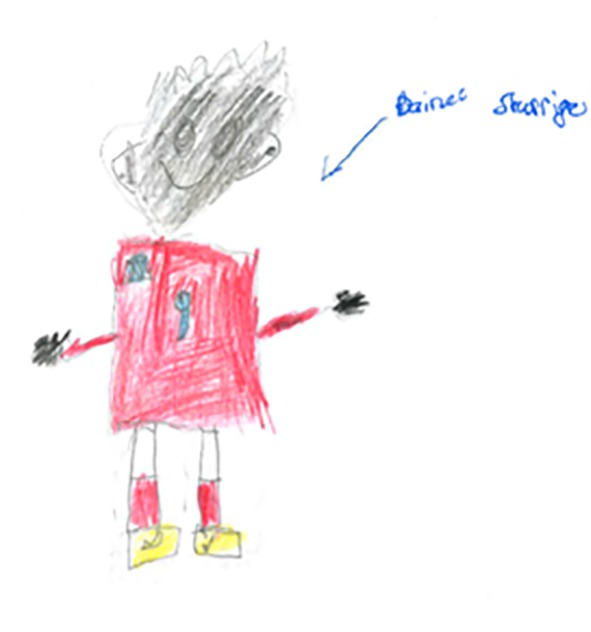
An English boy's drawing of a football player from England (shaded).

As mentioned previously, according to Koppitz (1968) shading is a sign of concern and based on her scoring scheme no score is given if there is a "deliberate shading of whole face or part of it, including "freckles," "measles," etc. An even, light shading of face and hands to represent skin colour is also not scored. However, most studies suggest that children normally do not shade their human figure drawings even among non-Western children. For example, Di Leo (1983) argued that both black and white children simply draw a contour for the face and do not colour it to show the skin colour. In a mega cross-cultural study Dennis (1966) asked children from Cambodia, Greece, Iran, Israel, Japan, Lebanon, Mexico, Sweden, Taiwan, Turkey, USA and West Germany to draw a man and found that the majority drew a white male figure in Western clothing. Pfeffer and Olowu (1986) found that Nigerian children, mean age 8, do not use dark colours even when they rarely use colours. Papadakis-Michaelides (1989) found that among the 3200 figures drawn by 1600 children from five cultural backgrounds in the UK (English, Hindu, Muslim, Sikh and West Indian) aged 3 years 6 months to 11 years 6 months only one figure was dark skinned. Thus it appears that even when children are aware of different skin colours they tend to draw a figure that looks like a white person. In the present study the results of analyses of the shading of the human figures showed that children from England more frequently drew human figures that were shaded than Iranian and Brazilian children, in spite of the fact that the majority of people in Iran and Brazil do not have a white Western appearance. Indeed it was also noted that male participants from England produced more shaded drawing than did their female counterparts, possibly because they were using more a model player to draw who happened to be of a non-Western origin (see Figure 4). It thus seems to be the case that when children have an external model in mind and the person to be drawn is non-white they do make use of shading in their drawings. This gives rise for future research to examine why children from England seem to be more sensitive to issues of skin colour (and race) than Brazilian or Iranian children. The study by Deaver (2009) is a good example of looking at difference in terms of race, gender and ethnicity from an intersectional analysis perspective, the social and cultural differences of the individual rather than a holistic interpretation of children’s drawings. Indeed children from England seem to be more conscious of different ethnic backgrounds and skin colours than do children from Iran and Brazil.

### Additional Observations

Whilst the main focus was to analyse the key factors mentioned previously, there was a noticeable feature about the gender of the player drawn. Whilst female football is running alongside male football in all three countries, with the only reservation being that women are obliged to play with strict dress code in Iran (Pfister 2005), 100% of the boys drew male players and only a very small percentage of girls (less than 5%) drew a player looking feminine. This seems to support the stereotypes that often exist about gender roles and football being seen as a masculine activity. In a study, reminiscent of the present study, Chambers (1983) examined the drawings of several thousand primary school children in three countries (Canada, Australia and USA) when the target to be drawn was a scientist. It was found that all of the boys drew a male looking scientist and only a very small number of girls drew a female looking scientist. Such gender stereotypes seem to have found support in relation to football in the present study and have implications when analysing the impact of possible stereotyping when evaluating the gender of drawings and the gender of the child.

## Summary and Implications

In the context of drawing a football player by children from three continents, five key factors that appear to play a significant role when assessing children's human figure drawings in educational, therapeutic and clinical settings were examined: namely size of the human figure, whether or not it is a profile, showing action or static, drawn in a context, and the inclusion of shading.

The results showed a highly significant main effect for cultural differences with Brazilian children showing marked differences compared to children from England and Iran in the size of the figure, depicting more action, including more context in what is drawn and drawing more profiles than full face. On the subject of shading, children from England, particularly males, drew more figures of players who had dark skin rather than white. What was interesting to note in the above findings was that when the child was given the opportunity to draw a topic of great interest, the smaller size was not necessarily a sign of dislike but perhaps a cultural factor. Similarly producing a profile was not a sign of being withdrawn but a sign of interest in the sport. Most significantly the usually reported gender differences in human figure drawings often interpreted in developmental terms were not found in the present study. Gender difference only manifests itself in shading by children from England, perhaps a sign of individual cultural and ethnic differences within a multi-cultural society. Thus rather than making fast decisions about a drawing, it is important to consider cultural differences, and whether or not the child had the opportunity for expression of their full potential by providing a context upon which they could flourish and for the drawing to be seen as a sign of interest in the topic and even as an indication of manifesting potential talents.

### Suggestions for Further Research

One factor that one could have incorporated in the present study is a more objective measure of level of interest in sport and football taken from all the participating children and to examine how it may relate to the level of detail included in their drawings. This could be implemented in a follow-up research, indeed emphasizing on the issue of talent, children’s drawing may also be looked at as a predictive tool for their future sporting activity. It may be the case that children who portrayed more action rather than a static picture of a football player may also demonstrate greater skills in playing football. Perhaps this would open a host of interesting dimensions for assessment of children's human figure drawings of different sporting activities as a key to predicting future sporting talents.
